# Castration for Rape in Kansas

**Published:** 1897-08

**Authors:** 


### CASTRATION FOR RAPE IN KANSAS.

A bill has been introduced into the Kansas Legislature, referred to the Committee on Public Health and Hygiene, and by them reported back with the recommendation that it pass. It provides that every person who shall be convicted of rape, and every person who shall be convicted of incest, and every minister, clergyman, priest or teacher, having charge of any church or other religious body or school, who shall have illicit connection with any unmarried virgin female under 21 years of age of his charge or school; and every guardian of any female ward under the age of 18 years who shall defile her, shall be punished by imprisonment at hard labor for a period not less than five nor no more than twenty years, and in addition to such punishment shall be castrated.

In all cases where the defendant shall be convicted of any of the offenses specified in the foregoing section, the court shall, when pronouncing judgment upon the defendant, order that the prisoner shall, after the expiration of one year from the date of his arrival at the penal institution in which he is to be confined, and within eighteen months from the date of the sentence, be castrated by the medical officer of such penal institution; which order shall be a part of the final judgment in such case, and a certified transcript of which order and judgment shall be delivered with the prisoner to the principal officer of the penal institution in which the prisoner is to be confined.

In any case where the order for such operation has been carried into effect under the provisions of this act, and after two months from the time of such operation, the Governor may, if satisfied that the prisoner will lead a peaceful and orderly life, issue to such prisoner a parole or conditional pardon, to be valid only during the good behavior of such person.

If the bill should become a law it will be interesting. It will be noticed that the bill practically places seduction and incest in the same category as rape. It is to be presumed that in Kansas there will be no difficulty in defining the use of the word "defile" as applied to guardians and wards.—*Medicine.*

